# Seasonal Impacts of Particulate Matter Levels on Bike Sharing in Seoul, South Korea

**DOI:** 10.3390/ijerph17113999

**Published:** 2020-06-04

**Authors:** Hyungkyoo Kim

**Affiliations:** Department of Urban Design and Planning, Hongik University, 94 Wausan-ro, Mapo-gu, Seoul 04066, Korea; hkkim@hongik.ac.kr; Tel.: +82-2-320-1635

**Keywords:** particulate matter, bike sharing, seasonal impacts, Seoul, negative binomial regression

## Abstract

Bike sharing is increasingly attracting more riders in cities around the world for its benefits regarding the urban environment and public health. The public bike sharing program of Seoul, South Korea, first launched in October 2015, is now widely spread around the city and serves more than 27,000 riders daily. However, concerns are being raised as rising air pollution levels in Seoul, represented by particulate matter (PM) levels, in recent years may negatively discourage citizens from using bike sharing. This study investigates the impact of PM_10_ and PM_2.5_ levels on bike sharing use in Seoul and seeks to identify any seasonal differences. A series of negative binomial regression models, which take into account control variables like weather conditions and calendar events, are adopted to empirically measure the impacts. Results show that the PM levels yield statistically significant negative impacts (*p* < 0.01) on bike sharing use throughout the year. The impacts are particularly stronger in winter and spring, when the PM levels are higher. Findings suggest that PM levels may operate as driving factors for bike sharing use in addition to meteorological conditions like temperature, humidity, and precipitation.

## 1. Introduction

The use of bike sharing as a means of transportation in urban areas throughout the world grew over the last few decades. Following some of its seminal initiators of Amsterdam in the 1960s and Copenhagen in the 1990s [1,2], recent successful implementations in Paris, Lyon, and London dispersed the program to a larger number of cities around the world that seek new means of urban transportation. Statistics testify that the number of bike sharing programs in operation worldwide more than doubled since 2014 and exceeds 1600 as of 2018, where the total number of public-use bicycles worldwide also more than doubled to 18.2 million in the same period [3]. Ease of use, lower prices than other means, lack of congestion, and marriage with smart phone technologies make bike sharing ubiquitous. It attracts numerous intracity travelers, as well as large investments from both public and private sectors [4,5].

There are some criticisms, which include its benefits toward the privileged and marginalization of lower classes [6,7,8,9], little impact on reducing automobile dependency [10,11], and increasing concerns of rider safety [12,13,14]. Nevertheless, bike sharing is known to produce a wide range of benefits as research suggests. It benefits the environment by reducing car dependency so as to curtail greenhouse gas emissions [4,15,16,17,18]. Somewhat related to the first, it also relieves traffic congestion [19,20]. Another is its health contributions to the prevention of diseases and deaths by promoting physical activities [16,17,21,22,23,24]. A fourth is the lower injury and fatality outcomes compared to non-sharing environments [24,25]. Lastly, bike sharing realizes a multifaceted sharing economy by allowing cities to function more efficiently [26,27,28].

These benefits encourage cities to adopt and promote bike sharing. In doing so, key concerns of planners and policymakers often target removing barriers and developing solutions based on the varying contexts each city faces. Research is producing empirical findings to support them. One frequently addressed issue is the supply of relevant road infrastructure to create a safer and more predictable riding environment for bike sharing users [29,30]. Another would be the selection of locations for bike sharing stations to provide the most optimum level of service and access within constraints [31,32,33].

Another growing body of interest lies in the weather conditions that affect bike sharing users and present contextual differences. Several studies carried out in North America found that high temperatures and low quantity of snow positively affect bike sharing [34,35]. On the other hand, a Brisbane study reported no significant effects of temperature because of the city’s subtropical climate, while wind and rain yielded negative effects [36]. One study from Cork, a medium-size city in Ireland, argued that warmer temperatures and longer hours of sunshine promote longer trips [37]. Another from Daejeon, South Korea noted that temperatures over 30 °C, precipitation, humidity, and wind speed negatively affect bike sharing users [38]. These studies in general commonly found that bad weather conditions have adverse effects on using bikes as a commuting mode with some variances between contexts [39,40,41,42,43].

A potential issue that receives less interest but requires further investigation is the impact of air pollution on bike sharing or using bikes in general. Many studies looked into exposure of bicyclists to air pollution [44,45,46]. On the other hand, only a handful studies reported that higher air pollution levels yield a significant negative impact on bike sharing [47] and that air quality improvement has a significant positive impact [48].

Building on what was addressed so far, this study looks into Seoul, South Korea, a relatively less studied and unknown city, which enjoys rapidly growing bike sharing but at the same time suffers from chronic high levels of air pollution, including particulate matter (PM) levels [49]. Specifically, it empirically investigates how PM_10_ (particles with diameters equal to or less than 10 micrometers) and PM_2.5_ (particles with diameters equal to or less than 2.5 micrometers) levels affect bike sharing use in Seoul daily. It also examines whether seasonal differences exist in the effects and takes into consideration the wide variance found in Seoul’s annual PM levels. Findings of this study may help unveil the relationship between PM levels and bike sharing. It may also inform transportation and environmental planning and policymaking to achieve a more livable urban environment.

## 2. Case Context

**Figure 1:**
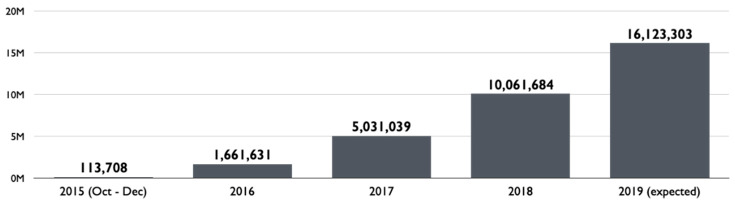
Annual bike sharing trips in Seoul. Data sources: Seoul Institute (https://www.si.re.kr/) and Seoul Facilities Corporation (http://www.sisul.or.kr/).

**Figure 2:**
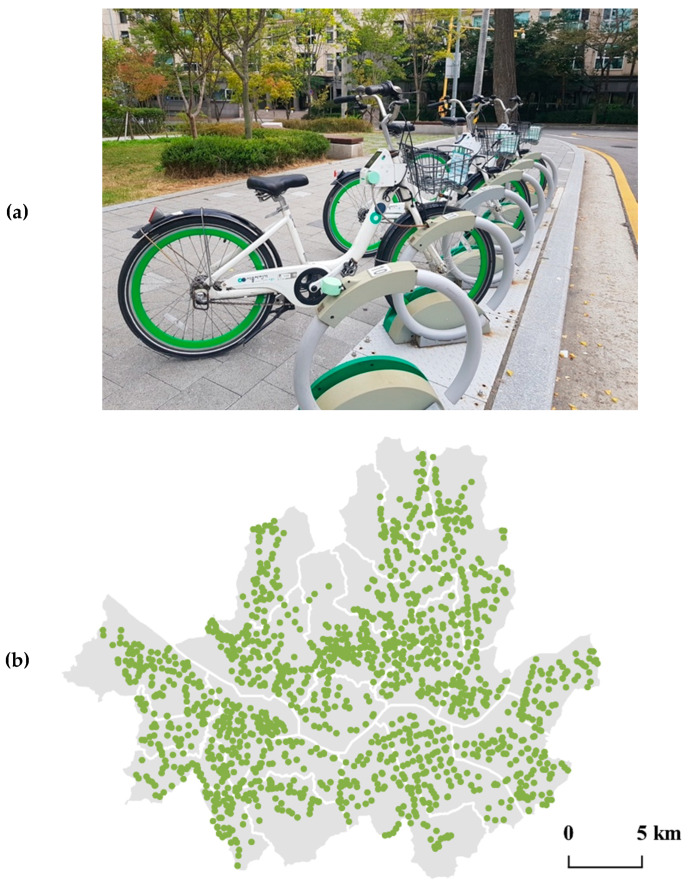
(**a**) A bike sharing station in Seoul; (**b**) locations of bike sharing stations in Seoul as of mid-2019.

After the successful launch of South Korea’s first bike sharing program in Changwon in 2008 [1,50], that of Seoul, or Ddareungi in local terms, started its service in October 2015. As Figure 1 illustrates, the program showed rapid growth over the past few years. From 2016 to 2019, the total annual number of trips almost decupled. In 2019, the number exceeded 16.1 million. This is comparable to that of New York City’s Citi Bike which served 17.6 million trips in 2018 [51] and also that of London’s Santander Cycles which accommodated 10.5 million in the same year [52]. Figure 2 presents that around 20,000 shared bikes and 1537 stations distributed throughout Seoul are in operation as of mid-2019.

**Figure 3:**
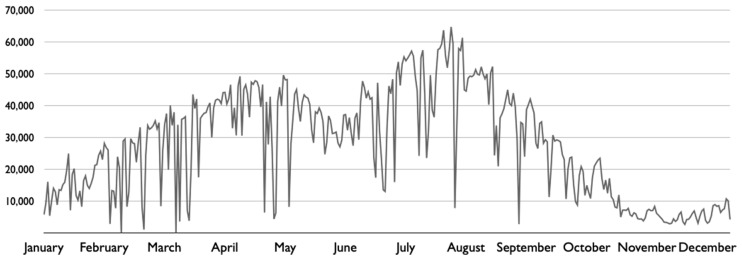
Daily bike sharing trips of Seoul in 2018. Data source: Seoul Open Data Plaza (https://data.seoul.go.kr/).

A closer look at Seoul’s bike sharing data presents clear seasonal fluctuations over the year. As Figure 3 shows, bike sharing use is the least in winter (December–February) with usually fewer than 20,000 trips daily, and it gradually increases through spring (March–May). During summer months (June–August), the use keeps increasing at first but decreases in August. In fall (September–November), bike sharing is the most used with up to more than 60,000 trips daily but decreases toward November.

**Figure 4:**
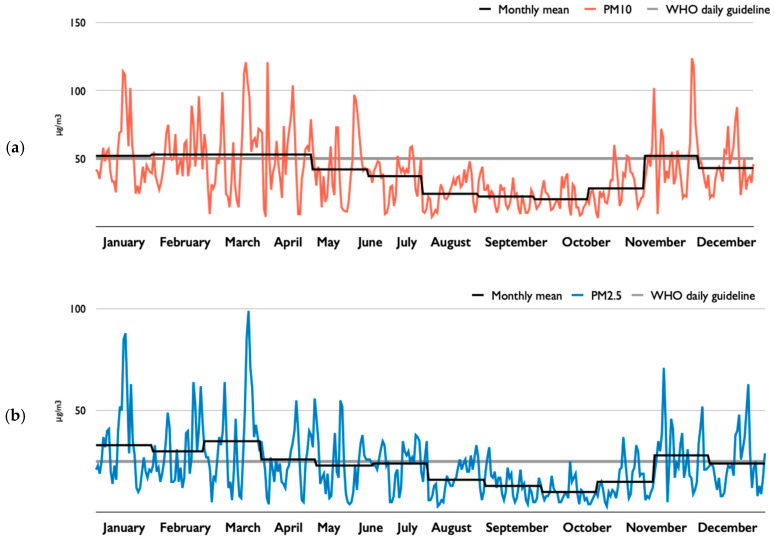
Daily mean particulate matter (PM) levels of Seoul in 2018: (**a**) PM_10_; (**b**) PM_2.5_. Data source: Seoul Metropolitan Government Air Quality Information (http://cleanair.seoul.go.kr/). Note: Seoul Metropolitan Government uses daily average concentrations of all stations in Seoul as the representative PM level of the city.

One critical issue that Seoul is currently facing is the serious air pollution levels. The city’s PM_10_ and PM_2.5_ levels maintained considerably high levels in the past few years. Especially in 2018, as Figure 4 illustrates, the number of days with PM_10_ levels higher than 50 micrograms per cubic meter, a World Health Organization (WHO) guideline for 24-h mean (annual mean: 20 micrograms per cubic meter), was 91, and that with PM_2.5_ levels surpassing 25 micrograms per cubic meter, also a WHO guideline for 24-h mean (annual mean: 10 micrograms per cubic meter), was 124. The mean PM levels of Seoul are significantly higher than those of major cities in the Organization for Economic Co-operation and Development (OECD) member countries [49]. In 2016, the annual mean PM_10_ level of Seoul was 40 micrograms per cubic meter, while that of Paris was 28, that of London was 23, that of Hong Kong was 34, and that of Melbourne was 19. Likewise, the annual mean PM_2.5_ level was 23 micrograms per cubic meter, while that of Paris was 16, that of London was 12, that of Hong Kong was 23, and that of Melbourne was 8 in the same year [53].

Exposure to PM is associated with a number of adverse health outcomes, including cardiovascular [54,55,56,57] and respiratory diseases [58,59,60] and various types of cancer [61,62,63]. Impacts on children [64], pregnant women [65], and the elderly [66,67] are reported to be more severe. In this sense, a recent OECD report warns that, by 2060, 1109 premature deaths per every one million people due to serious air pollution is expected in South Korea if further policy measures are absent [68].

Clear seasonal fluctuations characterize Seoul’s PM levels. As Figure 4 presents, some months of the year show higher PM_10_ and PM_2.5_ levels than others. Recent reports by local scientists present that heating, electricity production, and manufacturing, all of which heavily rely on fossil fuel-based energy in several neighboring countries during colder seasons, as well as the prevailing westerly winds, are responsible for up to 82% of Seoul’s PM concentration in high-PM seasons and around 30% in low-PM seasons [69]. They suggest that such fluctuations increase difficulty, inconsistency, and uncertainty in understanding Seoul’s PM levels and establishing relevant strategies and policies [70,71]. For these reasons, there is a strong need to recognize the seasonal differences when addressing Seoul’s PM levels.

## 3. Methods

So as to examine the effects of PM levels on bike sharing use in Seoul, this study adopts a range of variables as Table 1 summarizes. The dependent variables include daily total number of trips, total traveled distances, and total traveled times. Data were downloaded from Seoul Open Data Plaza (https://data.seoul.go.kr/), a publicly accessible website run by the Seoul Metropolitan Government.

The independent variables are daily mean PM_10_ and PM_2.5_ levels of Seoul. The local government operates 50 air quality monitoring stations (BAM-1020, Met One Instruments, Inc. (Grants Pass, OR, USA)) as of 2019. They are relatively evenly distributed throughout the city and are installed on publicly owned land. The government averages readings from each station to come up with a representative PM_10_ and PM_2.5_ level value for Seoul. The Seoul Metropolitan Government Air Quality Information (http://cleanair.seoul.go.kr/) provides related data.

Building on findings from the existing literature, a number of control variables are considered which are daily mean temperature, precipitation, mean wind speed, and mean humidity. Heavy rain (1: daily precipitation ≥15 mm; 0: daily precipitation <15 mm) and weekday (1: weekday; 0: Saturday, Sunday, and public holidays) are included as dummy variables so as to incorporate their categorial effects. Related data were acquired from the Korea Meteorological Administration (https://data.kma.go.kr/), a central government-level public agency responsible for collecting and sharing meteorological information in South Korea and providing forecasts. Due to data availability and completeness issues, the year 2018 at the daily level was used for analysis, since the number of missing values in data from 2016 and 2017 was considerably large enough to potentially distort results.

The three dependent variables are provided as mostly positive integers and a small number of zeros. This results in the need to adopt statistical models that effectively deal with count data. Furthermore, all the dependent variables present over-dispersion, meaning that the conditional variance exceeds the conditional mean, and they require using negative binomial regression for statistical analysis instead of Poisson regression because the confidence intervals are more likely to be narrower. Negative binomial regression is a highly robust statistical model favored in a wide range of fields, and it was proven to be highly effective in a number of existing studies that shared similar research interests [32,34,38,72].

So as to recognize the seasonal variations in Seoul’s PM levels and bike sharing trips, the regression models are applied to each of the four seasons, as well as for the whole year for reference. To address the problem of multiple hypothesis testing, Bonferroni correction is followed in the regression models. Moreover, since PM_10_ and PM_2.5_ levels are usually strongly correlated to each other, each regression model includes only one of the two as the independent variable. To complement and confirm the significance of the regression models, sensitivity analysis which includes seasonal dummy variables with a multiplicative interaction term is carried out (see Appendix A).

## 4. Results and Discussion

### 4.1. Results

Table 2 summarizes descriptive statistics of all variables, as well as frequencies of dummy variables, for each season. As for all seasons, an average of 27,560 trips were made daily, each traveling more than 125 million meters and 757 thousand minutes in total. The average PM_10_ and PM_2.5_ levels were 39.7 and 22.8 micrograms per cubic meter, respectively. In spring, the total number of trips and the traveled distances and times were smaller than the annual average. On average, 22,833 trips traveled around 107 million meters for 682 thousand minutes daily. The average PM_10_ and PM_2.5_ levels in spring were above the yearly average at 48.7 and 27.5, respectively.

Bike sharing in summer was used more frequently and longer. An average of 36,353 trips were made daily, and the total traveled distances and times exceeded 169 million meters and one million minutes, respectively. The average PM_10_ levels remained below 28, and those of PM_2.5_ remained at 17.8.

In fall, the numbers peaked when the number of daily trips exceeded 42 thousand, traveled distances neared 194 million meters, and traveled times reached 1.15 million minutes, while the average PM_10_ and PM_2.5_ levels were 33.3 and 17.5, respectively.

Lastly, in winter, the overall numbers for bike sharing use fell sharply as the average daily number of trips dropped to 9257. Total traveled distances and times in this season were 31.5 million meters and 182 thousand minutes, respectively, and PM_10_ and PM_2.5_ levels rose to 49.2 and 28.7, respectively.

It is noteworthy that the standard deviations of the PM_10_ levels in summer (12.8) and of the PM_2.5_ levels in summer (9.4) and fall (12.6) are smaller than other seasons. This may result in regression coefficients being insignificant because of lack of information.

Table 3 presents negative binomial regression results in estimating bike sharing use for all seasons using PM_10_ level data. In all three cases, the mean PM_10_ level presents significantly negative impacts (*p* < 0.01) on bike sharing use. Specifically, for a one-unit increase in the mean PM_10_ level, the expected log count of the number of trips decreases by 0.0034, traveled distances decrease by 0.0043, and traveled times decrease by 0.0041. Similar findings are shown with PM_2.5_, as Table 4 suggests. Again, the mean PM_2.5_ level yields significant negative influences (*p* < 0.01) on bike sharing use. The regression coefficients in estimating number of trips, traveled distances, and traveled times are −0.0080, −0.0094, and −0.0087, respectively. It can be interpreted that, throughout the year, bike sharing use in all three ways is negatively affected by PM_10_ and PM_2.5_ levels and that the latter shows larger impacts. With regard to the control variables, temperature presents significant positive impacts (*p* < 0.01) on bike sharing use, while precipitation, wind speed, and humidity exhibit negative effects (*p* < 0.01) in all cases.

Results for spring slightly differ. As Table 5 suggests, when estimating bike sharing use with PM_10_ level data, significant negative impacts (*p* < 0.1) are found for traveled distances and times but not for the number of trips. For a one-unit increase in the mean PM_10_ level, the expected log count of traveled distances decreases by 0.0029 and that of traveled times decreases by 0.0027. Table 6 presents comparable results for PM_2.5_. Significant negative impacts (*p* < 0.1) of the mean PM_2.5_ level are evident for traveled distances and times, and the regression coefficients are −0.0043 and −0.0041, respectively, as opposed to the number of trips. In sum, higher PM levels may not influence bike sharing trip numbers but clearly reduce the lengths of each trip distance- and time-wise. Among the control variables, temperature shows significant (*p* < 0.01) positive impacts, and precipitation and humidity present significant negative influences (*p* < 0.01) in the two models. The impacts of wind speed are no longer statistically significant.

Estimation results for summer show considerable differences. As Table 7 and Table 8 commonly suggest, PM_10_ and PM_2.5_ levels no longer operate as critical influencers on bike sharing use. Rather, temperature, precipitation, and humidity all yield significant negative impacts (*p* < 0.01). Results suggest that bike sharing users are more sensitive to the hot and humid weather conditions of summer and less affected by the PM levels, which are the lowest on average among the four seasons.

Fall shows relatively similar results to summer. Table 9 and Table 10 present that PM_10_ and PM_2.5_ levels do not show any clear impacts on bike sharing use. As the temperatures gradually drop in this season, higher temperatures seem to be significant positive influencers (*p* < 0.01), while humidity significantly reduces the use (*p* < 0.01). Impacts of other control variables are minimal.

Estimation results for winter are clearly different from the other seasons. As Table 11 presents, the mean PM_10_ level shows significant negative impacts (*p* < 0.01) on bike sharing use. Specifically, for a one-unit increase in the mean PM_10_ level, the expected log count of the number of trips decreases by 0.0152, traveled distances decrease by 0.0165, and traveled times decrease by 0.0152. PM_2.5_ also shows significant negative impacts (*p* < 0.01) on all three dependent variables, as found in Table 12. For a one-unit increase in the mean PM_2.5_ level, the expected log count of the number of trips decreases by 0.0174, traveled distances decrease by 0.0191, and traveled times decrease by 0.0175, demonstrating relatively larger impacts than PM_10_. As for the control variables, temperature generates significant positive impacts (*p* < 0.01) while precipitation generates significant negative impacts (*p* < 0.01) in both cases.

### 4.2. Discussion

For a more compact discussion of the results, Table 13 summarizes the coefficients of the mean PM_10_ and PM_2.5_ levels in estimating bike sharing use from each of the 10 regression models. Both PM_10_ and PM_2.5_ levels present significant negative impacts on the number of bike sharing trips, traveled distances, and traveled times in all seasons and winter, while controlling for meteorological conditions. In spring, the PM_10_ and PM_2.5_ levels show significant negative impacts on traveled distances and times, while no significant impacts are identified in summer and fall.

These results evidently demonstrate the seasonal differences that exist in the impacts of PM levels on bike sharing use in Seoul. In general, bike sharing users are largely affected by PM levels in high-PM seasons like spring and winter but not as so in low-PM seasons like summer and fall. Between spring and winter, the negative impacts of PM levels are more apparent and sizable in winter even though the mean PM_10_ and PM_2.5_ levels of the two seasons do not differ considerably from each other, as previously shown in Table 2. This may be attributed to the temperate weather conditions in spring, making bike sharing users relatively less concerned about air quality.

One noteworthy finding from the series of negative binomial regression results is the strong impacts of meteorological conditions. Similar to what previous studies identified [38,40,43], temperature was found to be statistically significant in all cases, and precipitation and relative humidity were statistically significant in a number of cases. Many of their regression coefficients presented larger impacts than those of PM_10_ and PM_2.5_ levels. Especially in summer, temperature, precipitation, and humidity strongly discourage bike sharing use, while PM levels do not make any difference. However, this does not indicate that the PM impacts are relatively minor but that they may also operate as key factors for bike sharing use in spring and winter in addition to meteorological conditions.

## 5. Conclusions

This study investigated how PM_10_ and PM_2.5_ levels affect bike sharing use in Seoul, South Korea, a city that experiences rapidly increasing use of bike sharing and high PM levels all year round. It also examined whether seasonal differences in the effects exist. A series of estimations using negative binomial regression models present that PM_10_ and PM_2.5_ levels negatively affect bike sharing use in winter and mostly in spring, when the PM levels are generally higher, as well as in all seasons, but show no significant impacts in summer and fall, when the levels are lower. In addition, significant impacts of meteorological conditions on bike sharing are clearly witnessed throughout the year.

There are several shortcomings of this study. It investigated only for one year due to data issues. This may weaken generalizability of the findings so as to be applied elsewhere or other times. Additional concerns, including personal preferences of bike sharing users the spatial variation of PM in Seoul, which may also affect bike sharing use, were not considered. The study did not also incorporate road congestion or safety concerns, both of which may considerably affect bike sharing use. Another shortcoming would be that the seasonal approach taken by this study may not successfully reveal critical conditions like very high-PM events which may yield different outcomes. Lastly, no distinction was made between work and non-work rides, which may yield differing outcomes when analyzed using hourly data, suggesting the need for further investigation. Future research may consider incorporating these issues into analysis, as well as considering adopting lag models to measure PM’s temporal impacts.

However, this study makes several contributions to the literature. It is one of the very first studies to empirically identify the impacts of air pollution in addition to meteorological conditions on bike sharing, and de facto the first for Seoul, taking the city’s seasonal fluctuations in PM levels and bike sharing use into account. Furthermore, it successfully unveils differences in the seasonal impacts, as well as those between the impacts of PM_10_ and PM_2.5_ levels. As a seminal research, it triggers further in-depth investigation in the near future at various levels using a wider range of information.

This study suggests some useful implications for planners and policymakers. It requires stronger omnidirectional measures to be adopted in cities to combat the high concentration of air pollutants. It also presents the need to actively address critical constraints like air pollution when promoting bike sharing and calls for seasonal approaches in developing solutions. Installing various infrastructures for bike lanes, such as buffers like half-walls or planters between car lanes [73] and street trees with high PM absorption capacities [74,75], would decrease concerns during high-PM seasons.

## Figures and Tables

**Table 1:** List of variables used in this study ^a^.

Variable		Unit	Data Source
Dependent variables	Total number of trips	-	Seoul Open Data Plaza
	Total traveled distances	meter	
	Total traveled times	minute	
Independent variables	Mean PM_10_ level	μg/m^3^	Seoul Metropolitan Government Air Quality Information
	Mean PM_2.5_ level	μg/m^3^	
Control variables	Mean temperature	°C	Korea Meteorological Administration
	Precipitation	mm	
	Heavy rain ^b^	1: precipitation ≥ 15; 0: precipitation < 15	
	Mean wind speed	m/s	
	Mean humidity	%	
	Weekday ^b^	1: weekday; 0: Saturday, Sunday, and public holidays	

Notes: ^a^ All variables are measured daily; ^b^ dummy variable.

**Table 2:** Descriptive statistics of variables by season.

Season	Variable	Mean or Frequency	SD	Min	Max
All seasons	Total number of trips	27,560	16,575	1036	64,644
	Total traveled distances (million meters)	125	86	3	390
	Total traveled times (thousand minutes)	757	544	19	2486
	Mean PM_10_ level (μg/m^3^)	39.7	23.1	6	124
	Mean PM_2.5_ level (μg/m^3^)	22.8	15.3	3	99
	Mean temperature (°C)	13.0	11.5	−14.8	33.7
	Precipitation (mm)	3.5	12.0	0	97
	Heavy rain ^1^	23	-	-	-
	Mean wind speed (m/s)	1.7	0.6	0.7	4.1
	Mean humidity (%)	57.5	15.1	23	97
	Weekday ^1^	261	-	-	-
Spring	Total number of trips	22,883	11,550	1036	43,468
	Total traveled distances (million meters)	107	62	3	252
	Total traveled times (thousand minutes)	682	419	19	1800
	Mean PM_10_ level (μg/m^3^)	48.7	27.6	7	121
	Mean PM_2.5_ level (μg/m^3^)	27.5	17.9	4	99
	Mean temperature (°C)	13.1	5.7	−0.7	23.2
	Precipitation (mm)	4.5	12.7	0	83
	Heavy rain ^1^	8	-	-	-
	Mean wind speed (m/s)	1.9	0.6	0.9	4.1
	Mean humidity (%)	59.2	16.3	23	97
	Weekday ^1^	64	-	-	-
Summer	Total number of trips	36,352	10,248	4357	49,519
	Total traveled distances (million meters)	169	54	18	275
	Total traveled times (thousand minutes)	1018	370	102	1784
	Mean PM_10_ level (μg/m^3^)	27.8	12.8	7	59
	Mean PM_2.5_ level (μg/m^3^)	17.8	9.4	3	38
	Mean temperature (°C)	26.6	3.7	20.2	33.7
	Precipitation (mm)	6.1	17.1	0	61
	Heavy rain ^1^	10	-	-	-
	Mean wind speed (m/s)	1.6	0.4	0.7	2.6
	Mean humidity (%)	65.1	12.8	39	95
	Weekday ^1^	66	-	-	-
Fall	Total number of trips	42,004	13,130	2728	64,644
	Total traveled distances (million meters)	194	80	8	390
	Total traveled times (thousand minutes)	1153	543	44	2486
	Mean PM_10_ level (μg/m^3^)	33.3	22.1	6	124
	Mean PM_2.5_ level (μg/m^3^)	17.5	12.6	3	71
	Mean temperature (°C)	14.1	6.4	1.9	25.5
	Precipitation (mm)	2.8	10.2	0	64
	Heavy rain ^1^	4	-	-	-
	Mean wind speed (m/s)	1.5	0.5	0.9	3.2
	Mean humidity (%)	59.2	12.4	27	94
	Weekday ^1^	65	-	-	-
Winter	Total number of trips	9256	6107	2640	24,620
	Total traveled distances (million meters)	32	22	8	99
	Total traveled times (thousand minutes)	182	122	46	575
	Mean PM_10_ level (μg/m^3^)	49.2	19.9	21	114
	Mean PM_2.5_ level (μg/m^3^)	28.7	16.5	8	88
	Mean temperature (°C)	−2.1	5.5	−14.8	11.5
	Precipitation (mm)	0.6	2.9	0	25
	Heavy rain ^1^	1	-	-	-
	Mean wind speed (m/s)	1.9	0.8	0.7	3.8
	Mean humidity (%)	46.3	12.1	26	87
	Weekday ^1^	64	-	-	-

^1^ Dummy variables.

**Table 3:** Estimation results for bike sharing use using PM_10_ level data in all seasons.

	Total Number of Trips		Total Traveled Distances		Total Traveled Times	
	*Coeff.*	*p*	*Coeff.*	*p*	*Coeff.*	*p*
Mean PM_10_ level	−0.0034 **	0.011	−0.0043 **	0.007	−0.0041 **	0.007
Mean temperature	0.0527 ***	0.000	0.0663 ***	0.000	0.0686 ***	0.000
Precipitation	−0.0192 ***	0.000	−0.0215 ***	0.000	−0.0231 ***	0.000
Heavy rain	−0.1121	0.613	−0.0531	0.842	0.0252	0.20
Mean wind speed	−0.2036 ***	0.000	−0.2213 ***	0.000	−0.2001 ***	0.000
Mean humidity	−0.0075 ***	0.004	−0.0094 ***	0.003	−0.0097 ***	0.001
Weekday	0.1157	0.071	−0.0376	0.626	−0.1341	0.065
*N*	365		365		365	
*2 Log Likelihood*	−7871.343		−14,064.148		−10,298.510	
*AIC*	7889.3		14,082		10,317	

** *p* < 0.05, *** *p* < 0.01. Note: Bonferroni-corrected *p*-values are applied to remove the problem of multiple hypothesis testing.

**Table 4:** Estimation results for bike sharing use using PM_2.5_ level data in all seasons.

	Total Number of Trips		Total Traveled Distances		Total Traveled Times	
	*Coeff.*	*p*	*Coeff.*	*p*	*Coeff.*	*p*
Mean PM_2.5_ level	−0.0080 ***	0.000	−0.0094 ***	0.000	−0.0087 ***	0.000
Mean temperature	0.0512 ***	0.000	0.0647 ***	0.000	0.0672 ***	0.000
Precipitation	−0.0203 ***	0.000	−0.0227 ***	0.000	−0.0243 ***	0.000
Heavy rain	−0.1283	0.558	−0.0684	0.795	0.0133	0.957
Mean wind speed	−0.2278 ***	0.000	−0.2479 ***	0.000	−0.2250 ***	0.000
Mean humidity	−0.0055 *	0.035	−0.0073 **	0.022	−0.0076 **	0.010
Weekday	0.1037	0.102	−0.0525	0.492	−0.1484 *	0.039
*N*	365		365		365	
*2 Log Likelihood*	−7862.803		−14,056.852		−10,291.406	
*Akaike information criterion*	7880.8		14,075		10,309	

* *p* < 0.1, ** *p* < 0.05, *** *p* < 0.01. Note: Bonferroni-corrected *p*-values are applied to remove the problem of multiple hypothesis testing.

**Table 5:** Estimation results for bike sharing use using PM_10_ level data in spring.

	Total Number of Trips		Total Traveled Distances		Total Traveled Times	
	*Coeff.*	*p*	*Coeff.*	*p*	*Coeff.*	*p*
Mean PM_10_ level	−0.0016	0.146	−0.0029 *	0.028	−0.0027 *	0.043
Mean temperature	0.0766 ***	0.000	0.0878 ***	0.000	0.0904 ***	0.000
Precipitation	−0.0332 ***	0.000	−0.0399 ***	0.000	−0.0388 ***	0.000
Heavy rain	0.2009	0.264	0.3107	0.142	0.2927	0.187
Mean wind speed	0.0191	0.719	−0.0197	0.752	−0.0202	0.757
Mean humidity	−0.0108 ***	0.000	−0.0128 ***	0.000	−0.0124 ***	0.000
Weekday	0.0648	0.309	−0.1177	0.116	−0.2431 ***	0.002
*N*	90		90		90	
*2 Log Likelihood*	−1793.18		−3334.478		−2430.358	
*Akaike information criterion*	1811.2		3352.5		2448.4	

* *p* < 0.1, *** *p* < 0.01. Note: Bonferroni-corrected *p*-values are applied to remove the problem of multiple hypothesis testing.

**Table 6:** Estimation results for bike sharing use using PM_2.5_ level data in spring.

	Total Number of Trips		Total Traveled Distances		Total Traveled Times	
	*Coeff.*	*p*	*Coeff.*	*p*	*Coeff.*	*p*
Mean PM_2.5_ level	−0.0025	0.128	−0.0043 *	0.030	−0.0041 *	0.048
Mean temperature	0.0757 ***	0.000	0.0862 ***	0.000	0.0889 ***	0.000
Precipitation	−0.0335 ***	0.000	−0.0403 ***	0.000	−0.0392 ***	0.000
Heavy rain	0.2059	0.251	0.3227	0.127	0.3061	0.167
Mean wind speed	0.0014	0.979	−0.0498	0.428	−0.0492	0.455
Mean humidity	−0.0104 ***	0.000	−0.0121 ***	0.000	−0.0118 ***	0.000
Weekday	0.0608	0.339	−0.1243	0.098	−0.2500 ***	0.002
*N*	90		90		90	
*2 Log Likelihood*	−1793.002		−3334.58		−2430.548	
*Akaike information criterion*	1811.0		3352.6		2448.5	

* *p* < 0.1, *** *p* < 0.01. Note: Bonferroni-corrected *p*-values are applied to remove the problem of multiple hypothesis testing.

**Table 7:** Estimation results for bike sharing use using PM_10_ level data in summer.

	Total Number of Trips		Total Traveled Distances		Total Traveled Times	
	*Coeff.*	*p*	*Coeff.*	*p*	*Coeff.*	*p*
Mean PM_10_ level	0.0009	0.633	0.0002	0.928	0.0006	0.792
Mean temperature	−0.0260 ***	0	−0.0332 ***	0	−0.0454 ***	0.000
Precipitation	−0.0178 ***	0	−0.0197 ***	0	−0.0212 ***	0.000
Heavy rain	0.0245	0.859	−0.0055	0.973	−0.0004	0.998
Mean wind speed	−0.0187	0.748	−0.0187	0.782	−0.0267	0.693
Mean humidity	−0.0077 ***	0.002	−0.0087 ***	0.002	−0.0105 ***	0.000
Weekday	0.1384 **	0.011	0.0190	0.763	−0.0486	0.441
*N*	92		92		92	
*2 Log Likelihood*	−1904.752		−3483.012		−2539.546	
*Akaike information criterion*	1922.8		3501		2557.5	

** *p* < 0.05, *** *p* < 0.01. Note: Bonferroni-corrected *p*-values are applied to remove the problem of multiple hypothesis testing.

**Table 8:** Estimation results for bike sharing use using PM_2.5_ level data in summer.

	Total Number of Trips		Total Traveled Distances		Total Traveled Times	
	*Coeff.*	*p*	*Coeff.*	*p*	*Coeff.*	*p*
Mean PM_2.5_ level	0.0007	0.789	−0.0003	0.925	0.0002	0.944
Mean temperature	−0.0262 ***	0	−0.0333 ***	0	−0.0456 ***	0.000
Precipitation	−0.0178 ***	0	−0.0197 ***	0	−0.0213 ***	0.000
Heavy rain	0.0217	0.875	−0.0089	0.956	−0.0038	0.981
Mean wind speed	−0.0167	0.773	−0.0170	0.801	−0.0246	0.715
Mean humidity	−0.0077 ***	0.001	−0.0087 ***	0.002	−0.0105 ***	0.000
Weekday	0.1394 **	0.010	0.0201	0.749	−0.0474	0.452
*N*	92		92		92	
*2 Log Likelihood*	−1904.901		−3483.011		−2539.607	
*Akaike information criterion*	1922.9		3501		2557.6	

** *p* < 0.05, *** *p* < 0.01. Note: Bonferroni-corrected *p*-values are applied to remove the problem of multiple hypothesis testing.

**Table 9:** Estimation results for bike sharing use using PM_10_ level data in fall.

	Total Number of Trips		Total Traveled Distances		Total Traveled Times	
	*Coeff.*	*p*	*Coeff.*	*p*	*Coeff.*	*p*
Mean PM_10_ level	−0.0003	0.853	−0.0017	0.315	−0.0020	0.281
Mean temperature	0.0367 ***	0	0.0531 ***	0	0.0597 ***	0.000
Precipitation	−0.0074	0.399	−0.0003	0.979	0.0042	0.717
Heavy rain	−0.5192	0.198	−0.8230	0.090	−0.9827	0.062
Mean wind speed	−0.0383	0.500	−0.0621	0.364	−0.0754	0.308
Mean humidity	−0.0122 ***	0	−0.0169 ***	0	−0.0189 ***	0.000
Weekday	0.0485	0.401	−0.0784	0.265	−0.1729 **	0.023
*N*	91		91		91	
*2 Log Likelihood*	−1930.961		−3493.132		−2571.092	
*Akaike information criterion*	1949		3511.1		2589.1	

** *p* < 0.05, *** *p* < 0.01. Note: Bonferroni-corrected *p*-values are applied to remove the problem of multiple hypothesis testing.

**Table 10:** Estimation results for bike sharing use using PM_2.5_ level data in fall.

	Total Number of Trips		Total Traveled Distances		Total Traveled Times	
	*Coeff.*	*p*	*Coeff.*	*p*	*Coeff.*	*p*
Mean PM_2.5_ level	0.0026	0.362	0.0005	0.883	0.0005	0.900
Mean temperature	0.0398 ***	0	0.0567 ***	0	0.0638 ***	0.000
Precipitation	−0.0045	0.617	0.0026	0.811	0.0075	0.527
Heavy rain	−0.5932	0.142	−0.8990	0.067	−1.0728 *	0.043
Mean wind speed	−0.0222	0.703	−0.0477	0.499	−0.0599	0.433
Mean humidity	0.0140 ***	0	−0.0182 ***	0	−0.0204 ***	0.000
Weekday	0.0519	0.372	−0.0777	0.272	−0.1718 **	0.025
*N*	91		91		91	
*2 Log Likelihood*	−1930.163		−3483.008		−2572.10	
*Akaike information criterion*	1948.2		3501		2557.6	

* *p* < 0.1, ** *p* < 0.05, *** *p* < 0.01. Note: Bonferroni-corrected *p*-values are applied to remove the problem of multiple hypothesis testing.

**Table 11:** Estimation results for bike sharing use using PM_10_ level data in winter.

	Total Number of Trips		Total Traveled Distances		Total Traveled Times	
	*Coeff.*	*p*	*Coeff.*	*p*	*Coeff.*	*p*
Mean PM_10_ level	−0.0152 ***	0	−0.0165 ***	0	−0.0152 ***	0.000
Mean temperature	0.1110 ***	0	0.1330 **	0	0.1285 ***	0.000
Precipitation	−0.1240 **	0.013	−0.1337 **	0.005	−0.1318 ***	0.003
Heavy rain	1.9941	0.101	2.1959	0.060	2.0818	0.051
Mean wind speed	−0.0304	0.650.	−0.0403	0.529	−0.0314	0.591
Mean humidity	−0.0062	0.298	−0.0094	0.103	−0.0080	0.125
Weekday	0.2774 **	0.005	0.1680	0.077	0.1607	0.064
*N*	90		90		90	
*2 Log Likelihood*	−1712.240		−3162.796		−2222.028	
*Akaike information criterion*	1730.2		3180.8		2240	

** *p* < 0.05, *** *p* < 0.01. Note: Bonferroni-corrected *p*-values are applied to remove the problem of multiple hypothesis testing.

**Table 12:** Estimation results for bike sharing use using PM_2.5_ level data in winter.

	Total Number of Trips		Total Traveled Distances		Total Traveled Times	
	*Coeff.*	*p*	*Coeff.*	*p*	*Coeff.*	*p*
Mean PM_2.5_ level	−0.0174 ***	0	−0.0191 ***	0	−0.0175 ***	0.000
Mean temperature	0.1012 ***	0	0.1224 ***	0	0.1187 ***	0.000
Precipitation	−0.1212 **	0.019	−0.1314 **	0.008	−0.1290 ***	0.005
Heavy rain	1.9035	0.128	2.1141	0.079	1.9939	0.070
Mean wind speed	−0.0747	0.289	−0.0893	0.188	−0.0761	0.219
Mean humidity	−0.0041	0.530	−0.0069	0.270	−0.0059	0.303
Weekday	0.2790 **	0.006	0.1691	0.083	0.1618	0.069
*N*	90		90		90	
*2 Log Likelihood*	−1715.924		−3167.469		−2226.91	
*Akaike information criterion*	1733.9		3185.5		2244.9	

** *p* < 0.05, *** *p* < 0.01. Note: Bonferroni-corrected *p*-values are applied to remove the problem of multiple hypothesis testing.

**Table 13:** Summary of regression coefficients of mean PM_10_ and PM_2.5_ levels.

Season	PM	Total Number of Trips	Total Traveled Distances	Total Traveled Times
All seasons	Mean PM_10_ level	−0.0034 **	−0.0043 ***	−0.0041 ***
	Mean PM_2.5_ level	−0.0080 ***	−0.0094 ***	−0.0087 ***
Spring	Mean PM_10_ level	−0.0016	−0.0029 *	−0.0027 *
	Mean PM_2.5_ level	−0.0025	−0.0043 *	−0.0041 *
Summer	Mean PM_10_ level	0.0009	0.0002	0.0006
	Mean PM_2.5_ level	0.0007	−0.0003	0.0002
Fall	Mean PM_10_ level	−0.0003	−0.0017	−0.0020
	Mean PM_2.5_ level	0.0026	0.0005	0.0005
Winter	Mean PM_10_ level	−0.0152 ***	−0.0165 ***	−0.0152 ***
	Mean PM_2.5_ level	−0.0174 ***	−0.0191 ***	−0.0175 ***

* *p* < 0.1, ** *p* < 0.05, *** *p* < 0.01. Note: Bonferroni-corrected *p*-values are applied to remove the problem of multiple hypothesis testing.

## Appendix A

**Table A1:** Estimation results for bike sharing using multiplicative interaction variables for seasons and PM_10_ level data.

	Total Number of Trips		Total Traveled Distances		Total Traveled Times	
	*Coeff.*	*p*	*Coeff.*	*p*	*Coeff.*	*p*
Mean PM_10_ level	−0.0031 **	0.011	−0.0033 **	0.034	−0.0026 *	0.068
(Mean PM_10_ level) × (Summer)	−0.0041 *	0.080	−0.0074 ***	0.009	−0.0086 ***	0.001
(Mean PM_10_ level) × (Fall)	0.0125 ***	0.000	0.0117 ***	0.000	0.0101 ***	0.000
(Mean PM_10_ level) × (Winter)	−0.0049 ***	0.002	−0.0071 ***	0.000	−0.0078 ***	0.000
Mean temperature	0.0534 ***	0.000	0.0668 ***	0.000	0.0685 ***	0.000
Precipitation	−0.0182 ***	0.000	−0.0206 ***	0.000	−0.0224 ***	0.000
Heavy rain	0.0308	0.870	0.0887	0.707	0.1488	0.504
Mean wind speed	−0.0975 **	0.033	−0.1187 **	0.038	−0.1119 **	0.036
Mean humidity	0.0192 ***	0.000	−0.0120 ***	0.000	−0.0118 ***	0.000
Weekday	0.1092 **	0.049	−0.0375	0.586	−0.1251 *	0.051
*N*	365		365		365	
*2 Log Likelihood*	−7760.657		−13,974.815		−10,202.575	
*Akaike information criterion*	7784.7		13,998.8		10,266.6	

* *p* < 0.1, ** *p* < 0.05, *** *p* < 0.01.

**Table A2:** Estimation results for bike sharing using multiplicative interaction variables for seasons and PM_2.5_ level data.

	Total Number of Trips		Total Traveled Distances		Total Traveled Times	
	*Coeff.*	*p*	*Coeff.*	*p*	*Coeff.*	*p*
Mean PM_2.5_ level	−0.0048 **	0.015	−0.0046 *	0.062	−0.0034	0.141
(Mean PM_2.5_ level) × (Summer)	−0.0058 *	0.096	−0.0108 **	0.012	−0.0126 ***	0.001
(Mean PM_2.5_ level) × (Fall)	0.0228 ***	0.000	0.0213 ***	0.000	0.0186 ***	0.000
(Mean PM_2.5_ level) × (Winter)	−0.0067 ***	0.007	−0.0098 ***	0.001	−0.0109 ***	0.000
Mean temperature	0.0551 ***	0.000	0.0688 ***	0.000	0.0706 ***	0.000
Precipitation	−0.0181 ***	0.000	−0.0206 ***	0.000	−0.0223 ***	0.000
Heavy rain	0.0344	0.857	0.0918	0.701	0.1523	0.499
Mean wind speed	−0.1104 **	0.019	−0.1337 **	0.023	−0.1246 **	0.023
Mean humidity	−0.0102 ***	0.000	−0.0118 ***	0.000	−0.0117 ***	0.000
Weekday	0.1126 **	0.043	−0.0349	0.615	−0.1227 *	0.058
*N*	365		365		365	
*2 Log Likelihood*	−7764.990		−13,979.984		−10,208.968	
*Akaike information criterion*	7789.0		14,004.0		10,233.0	

* *p* < 0.1, ** *p* < 0.05, *** *p* < 0.01.
